# Editorial: Oxytocin and metabolic dysregulation: from pathophysiology to pharmacotherapy

**DOI:** 10.3389/fendo.2025.1579937

**Published:** 2025-03-11

**Authors:** James E. Blevins, Pawel K. Olszewski

**Affiliations:** ^1^ Veterans Affairs Puget Sound Health Care System, Office of Research and Development Medical Research Service, Department of Veterans Affairs Medical Center, Seattle, WA, United States; ^2^ Division of Metabolism, Endocrinology and Nutrition, Department of Medicine, University of Washington School of Medicine, Seattle, WA, United States; ^3^ Biomedical, Molecular and Cellular Biology, University of Waikato, Hamilton, New Zealand; ^4^ Food Science and Nutrition, Twin Cities, University of Minnesota, Minneapolis, MN, United States; ^5^ Department of Integrative Biology and Physiology, University of Minnesota Medical School, Minneapolis, MN, United States

**Keywords:** obesity, glucose homeostasis, hypothalamic- pituitary-adrenal axis, stress, inflammation, food intake, thermogenesis, oxytocin

The many decades of searching for effective pharmacotherapies to combat obesity have brought mixed results. The excitement of the discovery of the fat-derived hormone leptin and its presumed potential for inducing weight loss, turned into disappointment when it became clear that obesity-associated leptin resistance prevents this hormone from lowering body weight in humans with obesity. More recently, the development of the long-acting glucagon-like peptide-1 (GLP-1) receptor agonist, semaglutide, has constituted one of the major pharmacotherapeutic advances. However, approximately one in six patients has been found to discontinue semaglutide treatment due to adverse events, primarily associated with gastrointestinal dysfunction. Thus, broadening the repertoire of drugs and multi-drug therapies, aimed at suppressing food intake and/or increasing energy expenditure, is required to further improve success rates of weight loss strategies.

One such candidate molecule is oxytocin (OT), traditionally well-recognized for its role in osmoregulation, and reproductive and social behavior. OT is an attractive therapeutic target to treat obesity because it reduces body weight in diet-induced obese (DIO) rodents, even in the presence of leptin resistance, and elicits cardioprotective effects in genetically obese rodents. This Research Topic highlights and expands our knowledge pertaining to the physiological and pharmacological effects of OT in the control of body weight, homeostatic/reward feeding, energy expenditure, pancreatic beta-cell health and stress.

Worth et al. highlights the involvement of paraventricular nucleus (PVN) OT neurons, including those targeting the bed nucleus of the stria terminalis (BNST), in food intake control. They report that chemogenetic activation of PVN OT neurons suppresses food intake in freely feeding mice. In contrast, inactivation of PVN OT neurons attenuates the response to meal-related satiety signal, cholecystokinin. Importantly, optogenetic stimulation of the PVN OT – BNST pathway results in sugar intake suppression; a finding which helps fill in the gap as to the identification of a neuronal relay that contributes to the role of endogenous OT in the control of sucrose intake and potentially other aspects of feeding reward.

The paper by Mendez et al. provides evidence suggesting that peripheral OT receptors (OTR) may be important in glycemic control. The authors found that OT stimulates insulin secretion in islets from control mice, but this effect is absent in mice with targeted OTR deletion from beta-cells. Similarly, OT reduces blood glucose levels in control mice with hyperglycemia and following a glucose tolerance test, but these effects are blocked in mice whose beta-cells did not express OTR. Furthermore, deletion of OTR from beta-cells leads to more marked hyperglycemia in response to streptozotocin relative to control mice, suggesting a protective role of beta-cell OTR. However, there is no alteration of beta-cell mass or apoptosis in low or high fat diet (HFD)-fed mice without beta-cell OTR but those on the HFD did display increased beta-cell proliferation.

Maejima et al. highlights the effects of the Japanese herbal medicine, Kamikihito (KKT), on body weight, food intake, sucrose solution intake and inflammation in both male and female middle-aged mice (which is a much needed departure from young rodent models in OT research), possibly by mimicking OT’s action. KKT ameliorated obesity-associated inflammation and reduces body weight and food intake in male mice. Though chow intake is not affected in female mice, KKT is effective at suppressing sucrose solution intake in females. Importantly, KKT decreases food intake and sucrose consumption in lean female middle-aged OT-deficient mice thereby raising the possibility that KKT may serve as an OTR agonist in the absence of native OT. Collectively, these findings suggest that KKT may be important in limiting age-related obesity and inflammation, in part by reducing food intake and/or intake of sugar-sweetened beverages, via OT-dependent mechanisms.

Uvnas-Moberg provides a comprehensive review of the complex interactions between OT and the hypothalamic-pituitary-adrenal (HPA) axis in the context of stress and the neural networks that contribute to OT’s anti-anxiolytic effects, including those that underpin stress-driven changes in appetite. These effects are mediated, in part, by neural relays from the supraoptic nucleus and PVN to, among others, the corticotropin-releasing hormone (CRH) neurons within the PVN to noradrenergic neurons in the locus coeruleus and CRH neurons in the amygdala. The authors expand on the temporal nature of the relationship between OT and the HPA axis, with both systems appearing to interact at birth and throughout the early-life period, leading to both immediate and long-term consequences.

Finally, Slattery et al. reports the effects of the combined effects of systemic OT and a beta-3 receptor agonist, CL 316243, on body weight and adiposity, food intake and brown adipose tissue (BAT) thermogenesis in DIO rats. These findings indicate that OT in combination with CL 316243 results in more pronounced reduction of body weight and adiposity relative to either monotherapy. These effects are mediated largely by OT-driven reductions of food intake and CL 316243-elicited increases in BAT thermogenesis. Given that OT has multi-faceted effects on homeostatic feeding and reward intake, it is not surprising that OT has also been found as an effective adjunct when used in combination with other compounds, namely the opioid antagonist, naltrexone, and GLP-1. In two separate studies, Head and Hsu found that naltrexone reduces body weight (1) or reduces consumption of a high-fat high-sugar meal (2) in humans and rodents. More recently, Maejima found that chronic administration of OT and GLP-1 reduces body weight in HFD-fed mice (3), thus further highlighting the potential use of OT as an adjunct with other drugs to treat obesity.

One important issue that the papers presented in this Research Topic have underscored is the contribution of peripheral OT in the control of food intake, energy expenditure, stress and glycemic control. While a plethora of studies support the importance of peripheral OTR in the control of food intake and body weight in rodents, it is clear that more clinical trials are needed to identify precise outcomes of peripheral OT treatment in humans (4, 5). The refinement of protocols, such as dosing duration, drug formulation, and the timing of administration, is necessary to allow the wealth of basic research evidence to be translated to the clinical practice.
